# Availability and Utilization of Automated External Defibrillators in New York State Schools

**DOI:** 10.3389/fped.2021.711124

**Published:** 2021-09-30

**Authors:** Milla Arabadjian, Stephanie Serrato, Mark V. Sherrid

**Affiliations:** ^1^Rory Meyers College of Nursing, New York University, New York, NY, United States; ^2^Hypertrophic Cardiomyopathy Program, Division of Cardiology, New York University Langone Health, New York, NY, United States

**Keywords:** automated external defibrillator, out of hospital arrest, sudden death, pediatric, school health

## Abstract

**Background:** Use of automated external defibrillators (AEDs) in out-of-hospital cardiac arrests (OHCAs) improve survival. Professional health organizations recommend that AEDs be available in crowded places, including schools but currently only 18 US states require them. Sudden cardiac arrest (SCA) research in the school-age population has largely focused on school sub-groups, leaving out the majority of US students and adults working in schools. New York State (NYS) has one of the largest student populations in the US. Our objective was to gain epidemiologic data on SCA across a variety of school levels and examine the availability and utilization of AEDs in a state that requires them.

**Methods:** This was an observational, cross-sectional study utilizing an electronic survey. We included NYS school nurses and collected electronic surveys in January-March, 2018. We analyzed demographic data of school characteristics, SCA occurrences and AED use and availability.

**Results:** Of 876 respondents (36.1% response rate), 71 (8.2%) reported SCAs, with 41 occurring in adults. AEDs were deployed in 59 of 71 (84.3%) events, 40 individuals had long-term survival. Most SCAs occurred in middle-schools. School size or number of AEDs/school had no bearing on short-term or long-term survival. AEDs were widely available in private schools, though this was not required by state law.

**Conclusions:** Our data suggest a need for more comprehensive examination of SCA in US schools. Research comparing the availability and utilization of school AEDs between states that do and do not require them is needed and may have important clinical and policy implications for SCA emergency preparedness in US schools.

## Introduction

Sudden cardiac arrest (SCA) is a public health concern with a severe impact on human health and well-being. In the United States, ~357,000 people experience out-of-hospital cardiac arrests (OHCAs) annually, and an estimated 70–90% of these individuals die before reaching the hospital (1). Cardiopulmonary resuscitation (CPR) and the use of an automated external defibrillator (AED) within minutes of OHCA can dramatically raise survival rates and improve neurological outcomes (2). For this reason, the American Heart Association recommends that AEDs be made available in public areas with high likelihood of SCA and high population density (3). Since at least 2004 interdisciplinary healthcare organizations have recommended that AEDs and AED programs be available in schools (4). Countries where AEDs are required in schools have shown success in mitigating SCA outcomes (2). Yet, in the US, only 18 states have legislation requiring AED placement in at least some of their schools and legislation regarding funding for these devices is highly variable (5, 6). One impediment to making AEDs available in all schools has been cost. However, the provision of AED machines and training would comprise a small portion of the per-student expenditures in the US, which are some of the highest in the world. Moreover, successful AED use has significant cost benefits in productivity and life gained (6–9).

Sudden cardiac arrest in children is relatively uncommon, though this phenomenon has not been comprehensively studied in US schools. The Resuscitation Outcomes Consortium found the incidence of SCA in children and adolescents to be 3.7 to 6.3 of 100,000 patient years (9). However, extrapolating SCA incidence in school- aged children from this data is difficult, as the authors defined children as those between 1 and 11 years old and adolescents between 12 and 19 years old. Further, the data included information from both the US and Canada, which have different school and healthcare systems (9). A systematic review examining OHCAs in schools included publications from the US and Japan, which also have markedly different school, public health and healthcare systems (10). In the US, the literature has largely focused on high schools and high school athletes (11, 12). One prospective observational study examined SCA incidence and risk of SCA among high school athletes and found the incidence of SCA to be 1.14 per 100,000 (13). An additional gap in the existing literature concerns adult SCA in schools, specifically SCA in school employees. In 2018, the last year for which there was available data, there were 3.7 million teachers and 3.3 million non-academic staff in US public schools alone, which comprised 2% of the 2018 US population overall (14, 15). It is unknown how many adults staff private schools in the US adding to this population.

New York State (NYS) requires AEDs in all public schools, though not in private schools (5). New York City (NYC) is the largest school district in the US, comprising of close to 1 million public school students (16) and 418,000 private school students (17). New York State (NYS) includes 2.6 million students in public school (18) and almost 800,000 students in private schools (17). The prevalence and incidence of SCA in NYS schools, among both adults and children, is unknown. The purpose of this study was 2-fold: to gain epidemiologic data on SCAs in NYS schools and to evaluate the availability and utilization of AEDs in schools. Given the paucity of data on SCAs in US schools overall, along with the variable state requirements on AED availability in schools, New York State data may have important practice and policy implications for future national implementation of these life saving devices.

## Methods

The study had an observational, cross-sectional design utilizing an electronic survey. Ethical study approval was obtained through our institution's Institutional Review Board. A waiver of consent was obtained. Data for this study is available with reasonable request. There was no patient or public involvement in this study. The inclusion criteria were school nurses who are actively practicing in NYS schools and we used purposive sampling in recruitment. We collaborated with the NYS Association of School Nurses, whose leadership disseminated the electronic survey on behalf of the research team, thus the researchers were blinded as to the individual identities of the study participants. School nurses are trained healthcare professionals who are involved in multiple aspects of school health, including emergency response (19, 20), and staff both public and private schools at all levels of primary education.

### Data Collection

Electronic surveys were sent to members of the NYS Association of School Nurses between January and March of 2018. The survey was designed to capture demographic characteristics of schools, including school size, locality and level of education provided. Primary school categories in NYS include elementary school, defined as grades Kindergarten to 5th grade (ages 5–11), middle school defined as grades 6 through 8 (ages 12–14), and high school defined as grades 9 through 12 (ages 14–18) (21). Combined schools included more than one school category. Additional variables were school nurse professional experience, availability of AEDs on premises, as well as SCA occurrence information, including survival. Short-term survival was defined as survival to transport to the hospital after EMS arrival and long-term survival was defined as survival to hospital discharge. There were 867 responses to the survey, a 36.1% response rate, which is consistent with the literature on response rates for online surveys (22).

### Statistical Analysis

Analysis included categorical descriptive statistics, frequency, proportions, and chi square analysis for evaluating associations between categorical variables. Bivariate logistic regression was used to examine independent predictors for short- and long-term survival. Missing data was excluded from analysis. Stata 16 software (23) was used. Results were considered statistically significant if *p*-value was <0.05 with 95% confidence interval.

## Results

### Baseline Characteristics

More than half of respondents, 481 (55.7%), were from New York City. Respondents worked primarily in public schools, 750 (86.7%). The majority of schools were either medium sized, with 250–500 students, 282 (32.6%), or large with over 500 students, 456 (52.8%). Respondents from elementary schools were most common, with 389 (44.9%) from public schools and 52 (6.0%) from private schools. More than half of respondents worked in schools that participated in interscholastic sports, 462 (53.4%). The majority of respondents indicated that AEDs were brought to interscholastic sport events, 277 (60%). Over a third of school nurse respondents had 5–15 years of professional experience, 301 (34.8%) (Table 1).

**Table 1:** Baseline characteristics.

**Baseline Characteristics**	***N***	**%**
**School nurse practice experience**		
<1 year	23	2.7
1–5 years	241	27.8
5–15 years	307	35.4
>15 years	296	34.2
**Type of School**		
Public	750	84.7
Private	116	13.4
Elementary	441	51.9
Middle School	100	11.6
High School	122	14.1
Combined (elementary ± middle ± high)	203	23.4
**Student body**		
<250	127	14.7
250–500	282	32.6
501–1,000	292	33.8
>1,000	164	19.0
**New York State regions**		
Capital Region	47	5.5
Central New York	58	6.7
New York City	481	55.7
Hudson Valley	78	9.0
Long Island	55	6.4
Southern Tier	34	3.9
Western New York	61	7.1
North Country	31	3.6
Finger Lakes	18	2.1
**Number of AEDs**		
None	47	5.4
1	264	30.5
2–4	436	50.4
≥5	119	13.7
**Participation in interscholastic sports**		
Yes	462	53.4
**AEDs brought to sports events**		
Yes	277	33.8

### SCA Events and AED Utilization

Seventy-one respondents (8.2%) reported SCA events in their schools with the majority being adult SCAs, 40 (57.1%). Only 5 events (7.0%) occurred in private schools. The majority of events were in public middle schools, 44 (66.2%), and these were split evenly between students and adults. The next highest SCA prevalence was reported by nurses working in combined public schools that house two or more educational levels, 11 (15.5%). Adults outnumbered students in this subgroup with 7 of the 11 occurrences (63.6%) (Table 2).

**Table 2:** Sudden cardiac arrest (SCA) events and AED utilization.

**Sudden cardiac arrest *N* = 71**	***N***	**%**
Student	30	42.9
AED deployed	59	84.3
Survival to EMS arrival and transport to hospital	50	84.7
Survival to discharge from hospital	40	80.0

Automated external defibrillators were deployed in 59 (84.3%) events. Of the 59 individuals with SCA where an AED was deployed, 50 achieved short-term survival, which was defined as survival to EMS arrival and transport to hospital. Of these, 40 individuals achieved long-term survival and resumption of regular activities. AED deployment was significantly associated with short-term and long-term survival, *p* = 0.018 and *p* = 0.005, respectively. School size, location, or number of AEDs in the schools were not associated with short- or long-term survival (Table 3). Long-term survival was associated with AED deployment regardless of other factors, such as the number of AEDs in the school, school size, or geographical location in NYS (OR 0.057, *p* = 0.035, 95% CI 0.004–0.816). AED deployment was also independently associated with short-term SCA survival regardless of school size or geographical region, (OR 0.823, *p* = 0.038, 95% CI 0.008–0.874). Half of respondents reported that their schools had 2–4 AEDs, 435 (50.2%), and a small minority of schools did not have an AED, 47 (5.4%). While AEDs are not legally required by NYS in private schools, the vast majority did have them, 114 (95.8%). 42 (5.4%) respondents from public schools reported no AEDs on their schools' premises; we did not inquire why in our questionnaire.

**Table 3:** Chi-Square associations between SCA survival and AED deployment, patient and school characteristics.

**Survival**	**School characteristics**	***p*-value**
Survival to EMS arrival and transport to hospital	AED deployment	0.018
	Number of AEDs in school	0.262
	School size	0.174
	School region	0.470
	SCA in adult	0.710
	Public school	0.224
Survival to hospital discharge	AED deployment	0.005
	Number of AEDs in school	0.838
	School size	0.516
	School region	0.325
	SCA in adult	0.707
	Public school	0.089

## Discussion

The study was novel in several ways. We gathered epidemiologic data on SCAs in schools in NYS in a comprehensive manner by surveying school nurses working at a variety of educational locales, including elementary and middle schools as well as private schools, which have been not been well-studied in the SCA school literature. School nurses are especially appropriate to provide this information as they work in a variety of school environments and are the healthcare providers in most schools where they serve the entire school population. The current literature has thus far largely focused on public high schools and student athletes (10–13). While more than half of US public high schools offer sports, student participation varies between 19 and 39% (24). Thus, the majority of US students do not participate in high school athletics. This is pertinent because childhood and adolescent non-athlete sudden death is actually more common than athlete sudden death (25). Hence SCA emergency response measures and AED availability policy should focus on schools overall and not preferentially on interscholastic athletics, as is the case where some states only require AEDs to be available only in schools that participate in interscholastic sports (6).

Even less is known about SCA in middle, elementary and private schools. The majority of SCAs reported in this study occurred in middle schools, underscoring the importance and need for further research of SCAs at every level of the educational continuum. Further, SCAs were also reported in private schools. While private schools in NYS are not legally required to have AEDs on premises, and most states that have provisions on AEDs omit private schools, most respondents reported that the devices were available to them. It is unclear if this is unique to NYS and merits further investigation.

Prevalence of adult SCA in school is also under-researched in general. Our study showed that adults had more SCA than students, which is expected given sudden death is more likely with advancing age (26). However, beyond examining AED utilization in this population within the school environment, further research may include study of factors that may impact sudden death in this adult population working in a chronically high stress environment (27, 28). This may identify sub-groups that could be at a higher risk for SCA, and thus identify potential avenues for intervention for SCA prevention, such as lifestyle modification of CAD risk. Other research may include evaluation of SCA response and AED intervention in adults and students to identify if there are differences among emergency response between these groups.

Several factors influence AED availability and utilization in schools. School nurse roles in AED education and preparedness vary in different school districts and are contingent on education department regulations, though they can be instrumental in the successful integration of SCA responses in schools due to their healthcare training. Harnessing the skills of school nurses to improve AED utilization would serve to improve SCA outcomes. This is especially important as currently, NYS does not require that all student facing staff is trained in cardio-pulmonary resuscitation (CPR) and AED use. Specifically, only coaches and athletic directors are required to have this training as a condition of their certification (29). Nationally, there are variable requirements relating to CPR/AED training for athletic coaches (30). Teachers are not universally required to be certified in CPR and AED use. Legislation in NYS is vague in requiring for there to be a cadre of staff volunteers who are trained in CPR/AED use (31). More recently CPR/AED use has been added to the curriculum of high school students as a condition to graduation (grades 9–12) (32, 33), which could improve out-of-hospital arrest response overall. However, instruction and standard vary across the US with differing implementation of the variable state requirements (34).

Cost had been cited as factor against universal implementation of AEDs in schools. However, there have been numerous studies outlining the relatively low costs of obtaining and maintaining AEDs in schools as well as training for staff, as well as the financial benefits in productivity of patient lives saved (35, 36).

## Limitations

This study had several limitations. First, we surveyed members of a professional organization, which might limit the generalizability of the results. However, to our knowledge, the NYS Association of School Nurses is the only school nurse professional organization in NYS, thus capturing a large majority of school nurses in the state. Second, our survey collected information regarding SCA events without a pre-determined timeframe, thus we are unable to report SCA incidence. We will further develop the questions of the survey in upcoming studies to capture more specific and detailed data regarding SCA occurrences. Thirdly, the response rate was only 36.1% and while this is consistent with response rates in the literature (21), we were limited in the number of reminders we were able to send, two, which differs from the recommended three reminders (37).

## Conclusion

Use of AEDs in OHCAs improves survival and long-term outcomes. Data on SCAs in school is largely limited to public high schools and high school athletics. However, SCAs occur at all educational levels and most US students do not participate in interscholastic athletics, though SCAs may be more common in non-athletes. Further, adults have more SCAs than students and this should be taken into account when evaluating school SCAs comprehensively. While this study examined AED availability and utilization in NYS schools, similar research is needed in other states, including those that do not have legal requirements regarding AEDs in schools. Policy implications include the wider adoption of AED availability requirements, which will contribute to improved SCA survival and outcomes.

## Data Availability Statement

The raw data supporting the conclusions of this article will be made available by the authors, without undue reservation upon reasonable request.

## Ethics Statement

The studies involving human participants were reviewed and approved by NYU Langone Health Institutional Review Board. Written informed consent for participation was not required for this study in accordance with the national legislation and the institutional requirements.

## Author Contributions

MA contributed in the conceptualization of the study, developing study protocol, obtaining regulatory approval, collecting and analyzing data, and drafting and editing the manuscript. SS provided had substantial contribution in concept development, data collection, and editing the manuscript. MS, as senior author, had a critically important role in guiding the study from conceptualization, to execution, to manuscript development, and including editing. All authors fulfill the authorship criteria set forth by ICMJE.

## Conflict of Interest

MA has received advisory panel fees from MyoKardia, Inc. MS has received consulting fees from Celltrion not relevant to this study, and as an unpaid consultant for MyoKardia, Inc. The remaining author declares that the research was conducted in the absence of any commercial or financial relationships that could be construed as a potential conflict of interest.

## Publisher's Note

All claims expressed in this article are solely those of the authors and do not necessarily represent those of their affiliated organizations, or those of the publisher, the editors and the reviewers. Any product that may be evaluated in this article, or claim that may be made by its manufacturer, is not guaranteed or endorsed by the publisher.
